# Tail shape and the swimming speed of sharks

**DOI:** 10.1098/rsos.231127

**Published:** 2023-10-11

**Authors:** Anthony S. Iliou, Wade Vanderwright, Lucy Harding, David M. P. Jacoby, Nicholas L. Payne, Nicholas K. Dulvy

**Affiliations:** ^1^ Earth to Ocean Research Group, Department of Biological Sciences, Simon Fraser University, Burnaby, British Columbia, Canada V5A 1S6; ^2^ Department of Zoology, Trinity College Dublin, Dublin D02 PN40, Ireland; ^3^ Lancaster Environment Centre, Lancaster University, Lancaster LA1 4YQ, UK

**Keywords:** shark, swimming speed, caudal fin aspect ratio, caudal fin, ‌ecology, morphological trait‌

## Abstract

Trait-based ecology is a rapidly growing approach for developing insights and predictions for data-poor species. Caudal tail fin shape has the potential to reveal much about the energetics, activity and ecology of fishes and can be rapidly measured from field guides, which is particularly helpful for data-sparse species. One outstanding question is whether swimming speed in sharks is related to two morphological traits: caudal fin aspect ratio (CFAR, height^2^/tail area) and caudal lobe asymmetry ratio (CLAR). We derived both metrics from the species drawings in *Sharks of the world* (Ebert *et al.* 2013 *Sharks of the world:* *a fully illustrated guide*) and related fin shape to two published datasets of (1) instantaneous swimming speeds (Jacoby *et al.* 2015 *Biol. Lett.* **11**, 20150781 (doi:10.1098/rsbl.2015.0781)) and (2) cruising speeds (Harding *et al.* 2021 *Funct. Ecol.* **35**, 1951–1959 (doi:10.1111/1365-2435.13869)) for 28 total unique shark species. Both estimates of swimming speed were positively related to CFAR (and weakly negatively to CLAR). Hence, shark species with larger CFAR and more symmetric tails (low CLAR) tended to be faster-moving and have higher average speeds. This relationship demonstrates the opportunity to use tail shape as an easily measured trait to index shark swimming speed to broader trait-based analyses of ecological function and extinction risk.

## 1. Introduction

A key challenge in trait-based ecology is to identify informative traits that can be readily measured for a wide range of individuals and species [1]. For example, there is a broad and applied interest in relating metabolic physiology to life histories, population dynamics and geographical distribution [2–4]. A current challenge is to identify morphological traits that relate to metabolic physiology and ecological function [3]. Two key metabolic morphological traits of interest to energy balance are gill slit height and measures of caudal fin shape (caudal fin aspect ratio (CFAR) and caudal lobe asymmetry ratio (CLAR)) which may be indicative of oxygen uptake capacity and expenditure, respectively. Requiem sharks with longer gill slits have a larger gill surface area, which in turn is positively related to metabolic rate within fishes [5]. More generally, respiratory surface area is closely related to metabolic rate, particularly in aquatic vertebrates [6–8].

Fin shapes can be measured from anatomically accurate drawings in natural history field guides [9,10]. The ground-breaking study by Sambilay *et al*. [9] identified a positive relationship between CFAR and swimming speed; however, this study was dominated by teleosts (*n* = 57 species, 119 cases) and it only considered *n* = 7 species of sharks (10 cases) [9], limiting our ability to generalize to this broadly threatened group of elasmobranchs. This study also pre-dated the ubiquitous access to electronic tagging which has yielded enormous insights into the swimming speeds of fishes [11,12]. Three decades on, it remains to be seen whether caudal fin shape is related to swimming speeds across a wider range of only shark species (subclass Elasmobranchii) which have more asymmetric heterocercal caudal fins.

We take advantage of two recent independent compilations of swimming speed estimates in wild sharks [11,12], comprising 26 species across 64 references [11], and 12 species, with 40 cases [12]. Here, we ask whether morphological traits (CFAR/CLAR) are related to swimming speed across a greater diversity of sharks.

## 2. Material and methods

### 2.1. Measurement of caudal fin aspect ratio and caudal lobe asymmetry ratio

**Figure 1.**
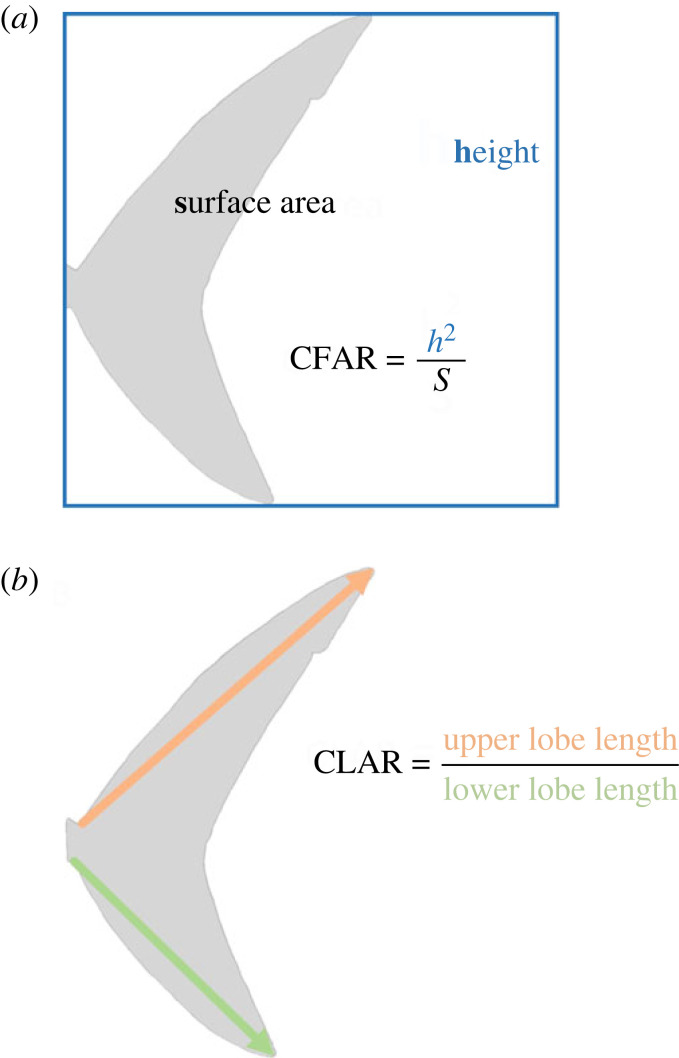
Measurements of caudal fin aspect ratio and caudal lobe asymmetry ratio. (*a*) The calculation of CFAR from tail height, its squared value shown in blue, and tail surface area, shown in light grey, with the formula shown (CFAR = height^2^/ surface area). (*b*) The calculation of CLAR using the upper lobe length, shown in orange, and lower lobe length, shown in green, using the formula CLAR = upper lobe length/lower lobe length.

CFAR measurements were taken from the anatomically accurate field guide, *Sharks of the world* [13]. Field guides were chosen as they are easily accessible, and as these illustrations are based on photographs of live individuals drawn by one illustrator (Marc Dando) in the same plane, they provide consistent measurements. Photos of each species drawing were imported into ImageJ, where the caudal fin height and lateral surface area were measured. CFAR was then calculated for each species as *A* = *h*^2^/*s*, where *h* is the height of the caudal fin and *s* is the surface area of the caudal fin (figure 1) [10]. We measured straight line distances from the nearest edge of each peduncle to the tip of the corresponding lobe and calculated CLAR as the ratio of upper to lower lobe length, such that a value of 1 means the lobes are symmetric (homocercal) and a value of 2 means the upper lobe is twice as long as the lower lobe (heterocercal; figure 1).

### 2.2. Swimming speed

Instantaneous swimming speeds and modal swimming speeds, in metres per second, were obtained from two previously published datasets compiled in two different ways [11,12]. The larger Jacoby *et al.* [11] literature review gathered estimates of instantaneous swimming speed from 64 primary sources spanning 26 shark species, via a variety of methods, including time/distance calculations and active ultrasonic tracking [11]. The smaller Harding *et al.* [12] primary research dataset is based on individuals captured by drumline, long line or angling, and tagged with biologging packages to directly measure speed in m s^−1^. They calculated modal speed representing the most common cruising speed of an individual, thus excluding bursts of activity that might be associated with foraging [12]. This study spanned 40 individuals from 14 shark species (electronic supplementary material, table S1). Additionally, 10 species were represented by more than one individual and arithmetic means were calculated to produce species averages.

This resulted in 40 speed measurements (*n* = 26 instantaneous, *n* = 14 modal; electronic supplementary material, table S1) for use in this study. We retained only sharks, excluding Atlantic bluefin tuna (*Thunnus thynnus*) and striped marlin (*Kajikia audax*), yielding 28 unique species, with an overlap of 12 species between both studies (*Hexanchus griseus, Notorynchus cepedianus, Carcharodon carcharias, Lamna ditropis, Galeocerdo cuvier, Carcharhinus amblyrhynchos, C. leucas, C. melanopterus, C. plumbeus, C. longimanus, Prionace glauca* and *Sphyrna lewini*). There was no overlap in individual sharks between datasets.

### 2.3. Statistical analysis

We asked to what degree swimming speed is positively related to CFAR measured from an anatomically accurate field guide, accounting for individual mass by fitting increasingly complex linear models and evaluated hypotheses using model selection and effect size estimation of parameters. Hence, we used a Bayesian framework to estimate the effect direction and effect size, rather than use a frequentist approach in which the *p*-value measures the probability that the null hypothesis (slope = zero) is true. A low *p*-value means that there is a low probability that the null is true and says little about how positive the slope is [14]. To evaluate the correlation between CFAR and CLAR, we used Pearson correlation coefficient. We evaluated the model fit and checked for linearity, homoscedasticity, independence, normality, and outliers using standard techniques. To account for the phylogenetic relatedness between species, we used a Bayesian framework to estimate parameters as implemented in the package ‘brms’. A full phylogenetic tree was used to account for the evolutionary history of traits between species (*n* = 28) [15]. The phylogenetic trees were extracted from https://vertlife.org/data/sharks/ and prepared in R using the ‘phytools’ and ‘ape’ packages. All analyses were done using R (v 4.2.0) in Rstudio [16].

## 3. Results

Instantaneous swimming speeds obtained from the Jacoby *et al*. dataset ranged from 0.09 m s^−1^ to 1.06 m s^−1^, and CFARs of the considered species ranged from 0.675 to 5.12, with a mean CFAR of 2.56. Modal speeds obtained from the Harding *et al*. [12] dataset, once averaged across species, ranged from 0.34 m s^−1^ to 1.06 m s^−1^. CFARs of the species within this dataset ranged from 0.939 to 4.74, with a mean CFAR of 2.66. The normal probability plot did not suggest any data points diverging from the *y* = *x* line and a graph of residuals versus fitted values showed no clear skewing and mostly followed a horizontal line. The variance inflation factors for the models did not exceed 2.3, showing no potential interactions between parameters. No values exceeded a Cook's distance of 1, and only one value, the whale shark, exceeded 0.5.

**Figure 2.**
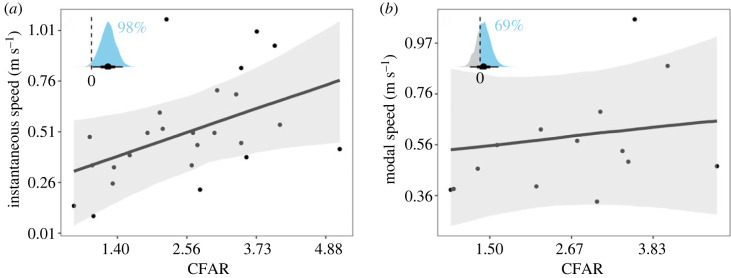
Swimming speed measurements as a function of caudal fin aspect ratio. (*a*) Instantaneous swimming speeds, with the slope coefficient represented by the dark grey line (0.12). 95% confidence intervals are represented by the grey shading, with points showing each shark species. (*b*) Modal swimming speeds, with the slope coefficient shown as the dark grey line (0.03). 95% confidence intervals are represented by the grey shading, with points showing each shark species. (insets) Posterior distributions for the model fit using either instantaneous speed–CFAR (*a*) or modal speed–CFAR (*b*) with the black dot representing the mean value, and zero shown with the dotted line. Values above 0 are shaded sky blue, and the percentage is shown in text next to the inset. All models shown include phylogeny.

Overall, species with higher swimming speeds have larger CFAR, especially for the larger Jacoby *et al*. [11] dataset, which had *n* = 26 species, compared to the Harding *et al*. [12] paper, which had 14 species (figure 2). Instantaneous swimming speed increased with CFAR in the larger Jacoby *et al*. [11] study (*β* = 0.12, 95% CI = 0.005 to 0.23, figure 2*a*; electronic supplementary material, table S2). The posterior distribution of the relationship between instantaneous swimming speed and CFAR was found to be positive for 98.1% of the iterations (figure 2 inset). Our model estimating instantaneous speed in relation to CFAR found the intercept evaluated at the mean CFAR value (2.56) was 0.50 m s^−1^. The model estimated that an increase in CFAR by one standard deviation (1.16) resulted in an increase in speed of 0.12 m s^−1^ (figure 2; electronic supplementary material, table S2).

In the Harding *et al*. [12] dataset, modal swimming speeds similarly increased positively with CFAR, but with a lower slope than the Jacoby *et al.* [11] study (*β=* 0.03, 95% CI = −0.086 to 0.16; figure 2*b*). The posterior distribution of the relationship between modal swimming speed and CFAR was found to be positive for 69.4% of the iterations (figure 2 inset). Our model estimating modal speed in relation to CFAR found the intercept evaluated at the mean CFAR value (2.66) was 0.60 m s^−1^. The model estimated that an increase in CFAR by one standard deviation (1.17) resulted in an increase in speed of 0.03 m s^−1^ (figure 2; electronic supplementary material, table S2).

Adding individual body mass to any of the two previous models did not improve the fit (looic values did not change by greater than two units; electronic supplementary material, table S3). Individual body mass also did not improve the model fit when individual modal speeds were not averaged within species (electronic supplementary material, table S3).

CLAR was negatively correlated to CFAR in both datasets (after eliminating the thresher shark, as its distinct caudal fin is not purely for locomotion) (Jacoby *et al*. Pearson's *r* = −0.73, Harding *et al. r =* −0.69; electronic supplementary material, figure S1*a*,*b*). Broadly speaking, the fastest sharks had high CFAR and the most symmetric tails and adding this ratio to the models did not significantly improve them but clearly this measure is worth exploring further (electronic supplementary material, figure S2*a*,*b*, table S4). Hence, species with high lobe symmetry (such as shortfin mako = 1.27, and whale shark = 1.37) had only moderately heterocercal tails and a considerable CFAR and swimming speeds. Whereas species with low lobe symmetry (leopard shark = 2) had the lowest CFAR and swimming speed.

## 4. Discussion

We show that CFARs measured from an anatomically accurate field guide are positively related to the swimming speeds of sharks, and CLARs are weakly negatively related to CFAR. Species with larger CFAR and more symmetric (homocercal) caudal lobes had, on average, faster swimming speeds. This generalizes a pattern well studied in teleost fishes [9] and our findings were robust to species identity, phylogenetic relationship, individual size and different measures of swimming speed (e.g. modal versus average). Next, we consider three issues: (1) ontogenetic change in swimming speed, (2) measures of swimming speed, (3) corroborating field-guide measures of CFAR.

### 4.1. Ontogenetic change in swimming speed

Here, we find an evolutionary allometry across species between caudal fin size and shape and two measures of swimming speeds. This may arise from an ontogenetic allometry where swimming speed likely increases with body size as species grow throughout their life, as seen in some species of fish [17]. Hence, one way of testing our interspecific finding would be to track the change in swimming speeds with changing tail size and shape within a species through ontogeny. An intraspecific analysis of swimming speed and fin shape would be a powerful complement to our general finding.

With increasingly sophisticated electronic tags, enabling more accurate and more frequent horizontal positional estimates that can be used to better measure distance travelled, we expect large advances in the analyses that are possible. Mainly, this will allow accounting for differences in methodology (e.g. acoustic tagging versus aquarium flume estimates or any other logging method with sufficient replicates) [18]. As more of these data accumulate and are archived in global databases [19], we recommend the calculation of ontogenetic allometries of swimming speed, using many different swimming metrics of the species. Ideally this would be augmented with measures of individual body mass. For large animals, body mass can be hard to measure, however, and therefore we recommend the use of length–weight conversion curves.

### 4.2. Measures of swimming speed

The relationship between both swimming speed metrics and CFAR was consistently positive, albeit with a slightly lower slope in the Harding *et al.* dataset. This dataset however had a much smaller sample size, and this may be the reason our confidence interval encompasses zero. There may also be differences in the fundamental nature of the different swimming speed measurements, and how they are potentially related to activity and metabolism. An average swimming speed over time overlooks behaviourally important hunting-related burst speeds [20]. A cruising speed measurement obtained by taking the most common speed measurement over time (i.e. modal) may give a different value than would be obtained had the same raw speed measurements been turned into a long-term average. We also acknowledge that there will be some noise within the relationship as some individuals will have different preferred speeds dependent on their activity at the time, for example, optimization for travelling versus foraging [21]. Swimming speeds per unit time measured from tagged animals will likely follow a power law frequency distribution offering opportunity to classify swimming species and time spent on different behaviours (cruising/travelling versus burst swimming associated with foraging). Hence, there may be merit in using a continuous measure such as the slope of the frequency distribution, as used to characterize Lévy flights [22]. Overall, as has been found in teleosts, we predict the fundamental relationship between caudal fin shape and a measure of swimming speed to remain, regardless of which measurement is used, where sharks with larger CFAR and more symmetrical lobes have overall faster swimming speeds. The next big leap forward in trait-based measures would be to consider other elasmobranchs, connecting the wing morphology of rays to swimming speeds and initial analyses of undulation to wing shape show much promise for this endeavour [23].

### 4.3. Future directions

We anticipate some concerns with using a field guide to interpret caudal fin morphology. In our experience, the placement of the caudal fin in a photograph can influence the CFAR whereby a more vertically placed or stretched upper lobe could artificially increase the perceived height and lobe ratio of our caudal fin. Our findings assume that each illustration was drawn accurately to represent how the caudal fin would behave while swimming [10]. We have confidence in the measurement accuracy of this field guide, as gill slit heights derived from this field guide were accurate when compared to field specimens [24] and similar measures of CFAR have revealed that more active species, with greater CFAR, had significantly greater gill surface area [10].

We have used readily available measurements from field guide illustrations in place of field or museum specimens, showcasing a promising and cheaper alternative. Additionally, this method provides a means for extrapolating to species for which such specimens are difficult to obtain (e.g. some deep-sea elasmobranchs). To further expand our analyses and corroborate our findings, we recommend collection of more caudal fin measurements from both field and museum specimens (including photos of live specimens), particularly to evaluate the unavoidable artefacts of preservations, such as shrinkage, as well as the challenge of flattening-out preserved specimens. However, there is a wider range of museum specimens readily available, that can be measured in a shorter space of time. These catalogues contain hard-to-collect species such as threatened, or uncommon species, so a combination of both fresh and preserved may be the best route to further corroborate these findings and give support for further research using accurate field guides as a resource. When it is possible to obtain measurements on live specimens, we also recommend the gathering of temperature data, as thermophysiology likely plays an important role in the relationship, which could help broaden our overall understanding between physiology and ecology [12,25,26].

Here, we have shown that swimming speed is positively related to CFAR (and weakly negatively related to CLAR) across four orders of sharks. This offers the opportunity for the development of an easily collected measure of activity and swimming speed of (mostly wild free-living) sharks. The challenge will be to expand this measure to rays and hence open the door to class-wide trait comparisons for the whole lineage.

## Data Availability

The data are provided in electronic supplementary material [27].
